# Geographical Constraints Are Stronger than Invasion Patterns for European Urban Floras

**DOI:** 10.1371/journal.pone.0085661

**Published:** 2014-01-22

**Authors:** Carlo Ricotta, Laura Celesti-Grapow, Ingolf Kühn, Gillian Rapson, Petr Pyšek, Frank A. La Sorte, Ken Thompson

**Affiliations:** 1 Department of Environmental Biology, University of Rome ‘La Sapienza’, Rome, Italy; 2 UFZ, Helmholtz Centre for Environmental Research, Department of Community Ecology, Halle, Germany; 3 German Centre for Integrative Biodiversity Research (iDiv) Halle-Jena-Leipzig, Leipzig, Germany; 4 Ecology Group, Institute of Agriculture and Environment, Massey University, Palmerston North, New Zealand; 5 Institute of Botany, Academy of Sciences of the Czech Republic, Průhonice, Czech Republic; 6 Department of Ecology, Faculty of Science, Charles University, Prague, Czech Republic; 7 Cornell Lab of Ornithology, Ithaca, New York, United States of America; 8 Department of Animal and Plant Sciences, University of Sheffield, Sheffield, United Kingdom; University of New South Wales, Australia

## Abstract

Understanding the mechanisms that affect invasion success of alien species is an important prerequisite for the effective management of present and future aliens. To gain insight into this matter we asked the following questions: Are the geographical patterns of species distributions in urban floras different for native compared with alien plant species? Does the introduction of alien species contribute to the homogenization of urban floras? We used a Mantel test on Jaccard dissimilarity matrices of 30 urban floras across the British Isles, Italy and central Europe to compare the spatial distribution of native species with four classes of alien species: archaeophytes, all neophytes, non-invasive neophytes, and invasive neophytes. Archaeophytes and neophytes are species that were introduced into Europe before and after 1500 AD, respectively. To analyze the homogenizing effect of alien species on the native urban floras, we tested for differences in the average dissimilarity of individual cities from their group centroid in ordination space. Our results show that the compositional patterns of native and alien species seem to respond to the same environmental drivers, such that all four classes of alien species were significantly related to native species across urban floras. In this framework, alien species may have an impact on biogeographic patterns of urban floras in ways that reflect their history of introduction and expansion: archaeophytes and invasive neophytes tended to homogenize, while non-invasive neophytes tended to differentiate urban floras.

## Introduction

Human activities are progressively weakening biogeographical barriers to dispersal, resulting in the spread and establishment of an increasing number of alien plant species. In some cases, alien species have become invasive, here defined as the rapid expansion of a species' distribution in a region outside of its historic range *sensu* [1] and [2]. Understanding the mechanisms that define successful introduction and invasion by alien species is an important prerequisite for the effective management of present and future aliens [3]. However, the impact of such species on the structure and composition of biological communities at broad geographical scales remains poorly understood despite intensive research in the last years [4]–[9].

Alien plant species vary substantially in the degree to which they are successful in their introduced environments. Invasion success in plant species depends on three primary components [10]. First, species' invasions are related to propagule pressure, as the invasion success greatly depends on repeated introductions, competitive strength, or mere chance, all of which is increased with increasing propagule pressure [11]–[16]. An additional factor related to propagule pressure is residence time or time since first introduction within the recipient region [17], [18]. Secondly, habitat invasibility plays an important role, as different communities, habitats or landscapes vary considerably in their level of invasion [15], [19]–[22]. Thirdly, successful invaders are often considered to possess traits that enable a species to effectively invade a new habitat, grow and reproduce [23], [24]. Single traits, however, have less power in explaining invasion success than combinations of several traits [25]. Species with more vigorous growth, higher fecundity and higher resource use efficiency were identified in a review by Pyšek and Richardson [26] as being more successful invaders. See also [27] for meta-analysis. Likewise, some reproductive, dispersal and cytological traits were shown to distinguish invasive alien species from non-invasive ones [28], [29], although other do not [27], [30].

In this framework, when documenting the ecological consequences of biological invasions, urban vascular floras are an informative focal group. On the one hand, given the greater availability of long-distance anthropogenic vectors of dispersal, cities serve as immigration sources with large pools of alien species, which can disperse into the surrounding, less disturbed, landscapes [31]. On the other hand, cities are often located in pre-existing biodiversity hot spots, thus harboring more native species than the surrounding landscapes [32], [33]. In addition, human disturbance in cities may provide a greater diversity of habitats than was originally present. For example, the presence of lime-based materials, such as concrete or mortar in many urban substrates may permit the establishment of calcicole species in areas previously dominated by calcifuges, while the ‘urban heat island effect’ may promote the establishment of species whose distributions are limited by the cooler temperatures of the surrounding areas [34].

Consequently, urban floras are generally rich both in native and alien species. This makes cities a suitable subject for comparing the coarse-scale geographical distribution of native and alien species.

The very different origin of alien and native species' pools would imply that there should be different distribution patterns for both groups of species. Nevertheless, just as for native species, the distribution patterns of aliens are constrained by environmental conditions and resource availability. These constraints act as filters limiting persistence of aliens in unfavorable habitats, so a degree of congruence with the native species is to be expected. As the species' distribution patterns are closely related to the concept of species' turnover or beta-diversity, we will also explore the effect of alien species on the floristic homogenization of the invaded regions. One hypothesized effect of plant invasions is that alien species should homogenize the invaded biotas, making these more floristically similar to each other. This can occur either by replacing local native species with widespread invasives *sensu* [35], or by adding widespread invasives to existing floras, thus increasing species richness [36]. However, evidence for floristic homogenization by aliens on a regional scale is still controversial. A number of studies on European regions [10], [33], [37], [38] show that alien species have had different impacts on species' turnover based primarily on residence time. Archaeophytes (species introduced before AD 1500) have had enough time to disperse and colonize large regions of Europe, thus promoting floristic homogenization; see also [18]. In contrast, neophytes (species introduced after AD 1500) usually show the opposite effect. A similar pattern was observed in North America: alien species originating from within North America were associated with homogenization whereas alien species originating from outside North America exhibited differentiation effects [39], [40].

To gain insight into this matter, we used contrasting historical, geographical and ecological perspectives provided by different residence times and invasion success of alien species for comparing their geographical patterns with corresponding patterns for native species in 30 European urban floras. We asked the following questions: i) Is the geographical pattern of species' distributions in urban floras different for native compared with alien plant species? ii) Does the introduction of alien species contribute to homogenizing the urban floras?

## Materials and Methods

For the purposes of this study, we analyzed 30 European urban floras. The database is composed of 10 floras for cities of the British Isles, 10 floras for cities of mainland Europe between 49° and 53° N latitude (hereafter simply mainland Europe), mainly located in Germany and the Czech Republic, and 10 floras for cities of Italy (Table S1). All cities of the British Isles and mainland Europe are located within the temperate deciduous forest biome [41], while the Italian floras are mainly representative of the Mediterranean climate zone.

The floras of individual cities included only spontaneously occurring species, excluding those kept only in cultivation or planted in public areas. For each flora, all varieties and subspecies were combined into single species, while microspecies were combined into their taxonomic aggregate species. For the floras of mainland Europe, the taxonomic nomenclature was standardized using TaxonScrubber (http://www.salvias.net/pages/taxonscrubber.html). For Italy and the British Isles, taxonomy was updated using [42] and [43], respectively. This resulted in a total of 3584 species for mainland Europe, 1612 species for the British Isles and 2097 species for Italy.

Each species was then classified as alien or native, where native species are defined as those that evolved or arrived in the study region before the Neolithic period or apparently arrived after that period independent of human activity [44]. According to their time of introduction alien species in mainland Europe and the British Isles were further classified as archaeophytes and neophytes. This classification system is widely used in western and central-European floras [45]–[47] and basically reflects the transition from regional to global origins of alien species in Europe. Archaeophytes are typically weeds of arable land introduced into Europe before AD 1500 primarily from the Mediterranean basin and south-eastern European steppes and are usually associated with rural environments and intermediate levels of human impact [48], [49]. In contrast, neophytes were introduced into Europe mainly from North America and Asia after the discovery of the New World, which marked the beginning of a new historical period of biotic interchange with expanding agriculture, industry and commercial exchanges.

The classification of urban plant species into natives, archaeophytes and neophytes is not necessarily consistent across Europe reflecting differences in the place of origin and time of introduction [50]. For instance, species that are native to a given region of Europe could be classified as archaeophyte or neophyte in a different region depending on their time of arrival, such as *Arrhenatherum elatius*, *Salvia officinalis* or *Thymus vulgaris*, which are natives to south-western Europe and archaeophytes (or neophytes) in central and northern Europe. Therefore, to provide a consistent classification scheme in which each species is identified by only one category, for the cities of mainland Europe we used the approach described by [37]. Species that were not designated exclusively as natives were classified as archaeophytes if they were identified as archaeophytes in at least one flora. Likewise, species were classified as neophytes if they were not identified as archaeophytes in any flora and were designated as neophytes in at least one flora. In doing so, aliens are ranked higher than natives because the alien status implies the ability to become established outside of the species' place of origin. Also, among aliens, archaeophytes are ranked higher than neophytes because of their earlier time of introduction into new regions outside their historical range [37]. As the Mediterranean region is one of the major sources of archaeophytes for central Europe, it is difficult to tell whether a species in the cities of southern Italy is native or archaeophyte due to the extremely long and intense history of land use in these regions. Therefore, for the Italian floras, only plant species introduced after AD 1500 (i.e. neophytes) were considered.

Based on their invasion status *sensu* [1] and [2], and primarily related to their rates of spread, neophytes were further classified as invasive and non-invasive, the latter group comprising species that occur as casual or naturalized plants, which are nevertheless not invasive. For cities in the British Isles all neophyte species occurring in Britain and Ireland in more than 25% grid cells of the PlantAtt database [51] were classified as invasive. For Italian cities we followed the nomenclature of the Italian checklist of alien plants [52]. For cities in the Czech Republic, we used invasive status ascribed to species in the national checklist of alien plants [53]. For cities in Germany, invasive neophytes were identified based on expert opinion (Kühn, unpublished). Finally, to provide a consistent classification of invasive and non-invasive neophytes across mainland Europe, we classified as invasive all species that were identified as such at least once either in Germany or in the Czech Republic.

To compare the distribution of native species with that of the alien species' groups (archaeophytes, all neophytes, invasive neophytes and non-invasive neophytes) we first calculated pairwise dissimilarity matrices among all urban floras for each species group using the index of Jaccard 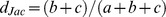, where *a* is the number of species present in both floras, *b* is the number of species present solely in the first flora (and absent from the second flora), and *c* is the number of species present solely in the second flora. In order to highlight possible differences between cities of mainland Europe, Italy and the British Isles, the calculation of the dissimilarity matrices and all subsequent analyses were run separately for each region.

To determine whether the spatial distribution of the native species was significantly different from that of the four groups of alien species we performed a Mantel test on the Jaccard dissimilarity matrices. This test basically consists of calculating the Pearson correlation coefficient between the elements (pairwise dissimilarities) in the triangular portions of two symmetric matrices. Because the elements of a dissimilarity matrix are not independent, *P-*values are obtained using a matrix permutation procedure (999 permutations, one-tailed test).

To test whether the introduction of alien species tends to promote floristic homogenization, we used the method proposed by [54]: the Jaccard dissimilarity matrices of the native species can be compared with the dissimilarity matrices of the alien species' groups by testing for differences in the average dissimilarity of individual cities from their group centroid in ordination space. First, we used the program PERMDISP2 [55] (http://www.stat.auckland.ac.nz/~mja/Programs.htm), to calculate the distance of each urban flora from its group centroid in principal coordinate space. Then, for each group of cities, we tested for differences in average dissimilarity from the group centroid between archaeophytes and natives, all neophytes and natives, invasive neophytes and natives, and non-invasive neophytes and natives, each with a paired *t-*test. *P-*values were obtained using pairwise permutation of the dissimilarities of individual cities from group centroids (999 permutations, two-tailed test).

## Results

**Figure 1:**
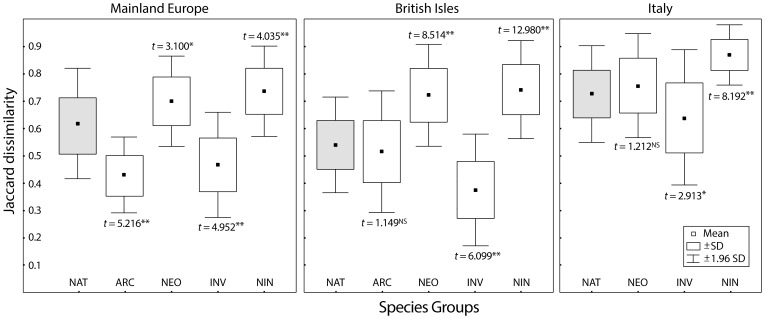
Box plots of the pairwise Jaccard dissimilarities between the urban floras of mainland Europe, the British Isles, and Italy. NAT  =  native species (in gray), ARC  =  archaeophytes, NEO  =  neophytes, INV  =  invasive neophytes, NIN  =  non-invasive neophytes. Values from permutation-based paired *t*-tests (999 permutations, two-tailed test) between native species and the three groups of alien species are shown. The significance levels are: **  = *P*<0.01; *  = *P*<0.05; NS  =  not significant at *P* = 0.05.

For all groups of cities the null hypothesis of no significant association between the spatial patterns of natives vs. alien species was rejected (Table 1), meaning that the distribution patterns of native and alien species are significantly associated with each other. Likewise, for all groups of cities, the mean Jaccard dissimilarity of the native floras was generally higher than the mean dissimilarity of the corresponding archaeophyte and invasive neophyte floras, being at the same time consistently lower than the mean dissimilarity of the neophyte and non-invasive neophyte floras (Figure 1). The high significance levels suggest that archaeophytes and invasive neophytes tend to promote homogenization of urban floras, while both neophytes and non-invasive neophytes tend to promote differentiation of urban floras.

**Table 1:** Results of the pairwise comparisons between the spatial distributions of native species vs. four groups of alien species (archaeophytes, all neophytes, non-invasive neophytes and invasive neophytes) for urban floras of mainland Europe, Italy, and the British Isles.

	Mantel correlation		
Species Groups	Mainland Europe	British Isles	Italy
Natives vs. Archaeophytes	0.388*	0.797**	—
Natives vs. Neophytes	0.468*	0.638**	0.819**
Native vs. Invasive Neophytes	0.567*	0.693**	0.852**
Natives vs. Non-invasive Neophytes	0.452*	0.609**	0.793**

The significance levels are: **  = *P*<0.01; *  = *P*<0.05.

## Discussion

As shown by the results of the Mantel test, the spatial pattern of all alien species' groups in the European urban floras is significantly associated with that of the native species. For instance, if the native floras of two cities are very different from each other, so are the corresponding alien floras. This suggests that, at this scale of analysis, the distribution patterns of native and alien species seem to respond to the same environmental drivers/filters [10], [50], [56]–[58]. Whether the similar distribution patterns of aliens and natives reflect underlying similarities of physiology, reproduction, dispersal and mortality is currently a matter of active debate [24], [59]–[62]. The lower level of significance associated with the Mantel coefficients for the floras of mainland Europe may be explained by the less consistent classification of urban species into natives, archaeophytes and invasive and non-invasive neophytes compared to Italy or the British Isles.

Within the stringent framework imposed by the observed similarity in their geographical patterns, alien species may have an impact on urban floras in ways which do reflect their invasion history and their rate of spread. The Jaccard dissimilarities calculated for the archaeophytes and invasive neophytes are on average lower than for the native species, while the dissimilarities between all neophytes and the non-invasive neophyte species are on average larger than for the native species. Therefore, archaeophytes and invasive neophytes tend to homogenize, while all neophytes and non-invasive neophytes tend to differentiate urban floras; see also [6], [10], [37].

We were unable to capture the rate at which (widespread) alien species substitute for (less widespread) natives, as this requires analyzing the same flora at different times (i.e. before and after invasion). Nonetheless, the higher Jaccard dissimilarities of the archaeophytes and invasive neophytes with respect to the native species tell us that the addition of both groups of alien species on native floras tend to exert a significant homogenizing effect. There are several possible reasons for this result. Archaeophytes are likely to exhibit homogenization because they are mainly species of limited environments such as arable fields [49], and thus have very similar and homogeneous habitats. Although most of the cities we considered contain very few remnants of agriculture, urban habitats are readily invaded from the surrounding areas [63], and so offer ‘refuge’ to species living outside the urban area in habitats of comparable types [64], [65].

In contrast, neophytes, and particularly non-invasive neophytes, probably have yet to reach their environmental limits [66], [67], reflecting instead the role of anthropogenic drivers in determining their pathways of introduction [68]. Furthermore, these anthropogenic drivers have constantly changed over the past hundred years causing idiosyncratic distribution patterns that are not yet in equilibrium [69].

Neophytes, as long as they are not invasive, tend to differentiate floras [70]. This is actually what one would expect at the beginning of an invasion process. Newly arriving alien species enter by a multitude of different pathways [71], being released intentionally, escaping from gardens, forests or agricultural fields, being unintentionally introduced along with commodities or along with a transport vector, or simply dispersing along longitudinal human infrastructure such as railroads, rivers and canals without any further anthropogenic assistance. In addition, each of these pathways has a plethora of entry points into a new region. Therefore, it is highly likely that species introduced into a new range will initially spread according to a more erratic spatial pattern, which will inevitably lead to short-term differentiation between urban areas [70].

As found in other studies [4], invasive species generally exhibit lower dissimilarities than natives or non-invasive neophytes. There are two potential explanations for this greater homogeneity. Invasives most likely have been in the occupied territory longer, since the probability of becoming invasive increases with residence time [72], [73]; however, there are also many examples of alien species with long residence times that are not invasive, e.g. [74]. Also, invasives may be species that have a very high rate of spread [1], [75]. Therefore, as they are able to sample more habitats in shorter time, invasive neophytes tend to homogenize the urban floras at least as well as, if not better than the archaeophytes, which have had several centuries or millennia to saturate available habitats. Fortunately, so far only a minority of alien species are that invasive [76] such that if we look at the impact of all neophyte species together, dropping the invasive/non-invasive classification, we find that, overall, neophytes tend to differentiate urban floras. To the best of our knowledge, this is the first study showing that invasive species are indeed having a homogenizing effect among European urban floras.

As a cautionary note, like with other sources of floristic data, the use of floras as baselines for understanding patterns of urban biodiversity has limitations. Such limitations are mainly related to inconsistencies in the way the data are collected. In this study, data were extracted from floras published over two to three decades by multiple researchers (see [41] and Table S1). During this period, urban floras have likely continued to change, including introductions of new alien species, together with the conventions used for sampling urban floras. These differences may contribute to the differentiation between cities observed in this study. One might also argue i) that the classification of neophytes into invasive and non-invasive species is not consistent among the studied regions, thus biasing the results obtained, and ii) that using geographical extent in classifying invasive species leads to some circular reasoning regarding biotic homogenization: in principle, any group of widespread species might be more similar between sites than any other with species of more constrained distributions. However, for all groups of cities, invasive species were defined based on available information and without reference to their abundances in the urban floras. Therefore, their homogenizing effect on urban floras is not an automatic outcome of the way invasive species were defined. The relevant point here is not the homogenizing effect of invasive species on urban floras. Although [77] used countryside survey data of British plant communities to show that it is possible to be invasive and yet, at the landscape scale, still relatively uncommon, the homogenizing effect of invasive species would be probably the same with every ecologically reasonable definition of invasiveness, as this concept is inevitably connected to homogenization. The relevant point here is rather the observation that the geographical pattern of invasive species is significantly associated to that of natives, thus setting a potential upper limit for the homogenizing effect of alien species.

To sum up, the increase in processes facilitating invasions, like international trade, travel, anthropogenic disturbance or climate change [69], [78]–[80], has led to the progressive mixing of biota from across the world with increasing rate of establishment of alien species, including invasive ones. Therefore, some degree of biotic homogenization is the inevitable result. However, as for native species, which differ in many respects, alien species are not a homogeneous group in themselves. Considering processes that are selectively filtered by humans, such as invasion pathways, one may find differences between different groups of alien species. On the one hand, our analysis gives support to a temporal aspect of homogenization coupled with the species' environmental requirements and rate of spread: the longer alien species are in their introduced location, the more likely it is that they will have a larger range [66], [67], [81]. On the other hand, the observed association between the spatial patterns of natives vs. all alien species' groups (including the invasive neophytes, which have the potential to exert strong impact on native communities [82]) suggests that, at least in urban areas, local environmental filters act as chief determinants of species' persistence in a given ecological space, setting an upper limit to biotic homogenization. Therefore, in spite of the increasing rate of alien invasion, as long as European cities remain environmentally distinct, a very intense homogenization of their floras is not to be expected.

## Supporting Information

Table S1 **Floristic data of 30 European urban floras used in this study with the geographical location, the total number of species, the number of species designated as native and alien and the number of alien species designated as archaeophyte (for mainland Europe and the British Isles only), non-invasive neophyte and invasive neophyte.**
(DOC)
