# Supplementary material for: Geographical Constraints Are Stronger than Invasion Patterns for European Urban Floras
Source: PLoS One. 2014 Jan 22;9(1):e85661. doi: 10.1371/journal.pone.0085661 (PMC3899062; doi:10.1371/journal.pone.0085661)
Supplement: Table S1 — Floristic data of 30 European urban floras used in this study with the geographical location, the total number of species, the number of species designated as native and alien and the number of alien species designated as archaeophyte (for mainland Europe and the British Isles only), non-invasive neophyte and invasive neophyte. (DOC) [file pone.0085661.s001.doc]

**Supporting Information**

**Table S1.** Floristic data of 30 European urban floras used in this study with the geographical location, the total number of species, the number of species designated as native and alien and the number of alien species designated as archaeophyte (for mainland Europe and the British Isles only), non-invasive neophyte and invasive neophyte. Sources: Poli Marchese et al. 1989 (Catania), Leporatti et al. 2001 (Chieti), Mele et al. 2002 (Lecce), Interdonato et al. 2003 (Messina), Banfi & Galasso 1998 (Milano), De Natale & La Valva 2000 (Napoli), Pirone & Ferretti 1989 (Pescara), Celesti-Grapow 1995 (Roma), Martini 2006 (Trieste), Verona et al. 2004 (Udine). The source literature used for the cities of mainland Europe and the British Isles can be found in Ricotta et al. 2009.

| City | Latitude/longitude | Number of species | | | | | |
| --- | --- | --- | --- | --- | --- | --- | --- |
|  |  | Total | Natives | Aliens | Archaeophytes | Non-Invasive Neophytes | Invasive Neophytes |
| *Mainland Europe* |  |  |  |  |  |  |  |
| Berlin, West (Germany) | 52°31’ N/13°24’ E | 955 | 397 | 558 | 240 | 273 | 45 |
| Brno (Czech Republic) | 49°12’ N/16°37’ E | 765 | 205 | 560 | 269 | 262 | 29 |
| Brussels (Belgium) | 50°50’ N/04°21’ E | 696 | 320 | 376 | 193 | 138 | 45 |
| Chemnitz (Germany) | 50°50’ N/12°55’ E | 837 | 409 | 428 | 207 | 179 | 42 |
| Halle an der Saale (Germany) | 51°28’ N/11°58’ E | 896 | 406 | 490 | 237 | 210 | 43 |
| Hannover (Germany) | 52°22’ N/09°44’ E | 782 | 423 | 359 | 199 | 128 | 32 |
| Leipzig (Germany) | 51°20’ N/12°23’ E | 1732 | 589 | 1143 | 327 | 764 | 52 |
| Plzeň (Czech Republic) | 49°43’ N/13°29’ E | 1014 | 520 | 494 | 235 | 207 | 52 |
| Prague (Czech Republic) | 50°05’ N/14°26’ E | 1856 | 952 | 904 | 347 | 504 | 53 |
| Warsaw (Poland) | 52°15’ N/21°00’ E | 1379 | 725 | 654 | 294 | 316 | 44 |
| *British Isles* |  |  |  |  |  |  |  |
| Birmingham (UK) | 51°29’ N/01°54’ W | 565 | 397 | 168 | 90 | 43 | 35 |
| Brighton (UK) | 50°49’ N/00°08’ W | 529 | 339 | 190 | 113 | 48 | 29 |
| Dublin (Ireland) | 53°20’ N/06°15’ W | 306 | 195 | 111 | 56 | 29 | 26 |
| Exeter (UK) | 50°43’ N/03°31’ W | 473 | 322 | 151 | 82 | 33 | 36 |
| Kingston upon Hull (UK) | 53°43’ N/00°20’ W | 696 | 414 | 282 | 138 | 107 | 37 |
| Leeds (UK) | 53°47’ N/01°32’ W | 410 | 291 | 119 | 60 | 29 | 30 |
| Leicester (UK) | 52°38’ N/01°08’ W | 563 | 366 | 197 | 105 | 55 | 37 |
| London (UK) | 51°30’ N/07°39’ W | 1147 | 605 | 542 | 172 | 321 | 49 |
| Plymouth (UK) | 50°22’ N/04°08’ W | 730 | 464 | 266 | 125 | 100 | 41 |
| Sheffield (UK) | 53°23’ N/01°28’ W | 1418 | 805 | 613 | 195 | 365 | 53 |
| *Italy* |  |  |  |  |  |  |  |
| Catania | 37°30’N/15°05’E | 246 | 226 | 20 | --- | 4 | 16 |
| Chieti | 42°21’N/14°10’E | 404 | 369 | 35 | --- | 11 | 24 |
| Lecce | 40°21’N/18°10’E | 312 | 281 | 31 | --- | 8 | 23 |
| Messina | 38°11’N/15°33’E | 270 | 236 | 34 | --- | 12 | 22 |
| Milano | 45°27’N/09°11’E | 984 | 832 | 152 | --- | 80 | 72 |
| Napoli | 40°50’N/14°15’E | 797 | 698 | 99 | --- | 45 | 54 |
| Pescara | 42°27’N/14°12’E | 387 | 342 | 45 | --- | 8 | 37 |
| Roma | 41°53’N/12°28’E | 1289 | 1127 | 162 | --- | 96 | 66 |
| Trieste | 45°38’N/13°48’E | 972 | 864 | 108 | --- | 43 | 65 |
| Udine | 46°04’N/13°14’E | 681 | 585 | 96 | --- | 37 | 59 |

**References**

Banfi E, Galasso G (1998) La flora spontanea della città di Milano alle soglie del terzo millennio e i suoi cambiamenti a partire dal 1700. Memorie della Società Italiana di Scienze Naturali e del Museo Civico di Storia Naturale di Milano 28: 283–388.

Celesti-Grapow L (1995) Atlante della flora di Roma. Roma: Argos Edizioni.

De Natale A, La Valva V (2000) La flora di Napoli: I quartieri della città. Webbia 54: 271–375.

Interdonato M, Hruska K, Villari R (2003) Research on the urban flora of Messina. Annali di Botanica 3: 106–116.

Leporatti ML, Pavesi A, Massari G (2001) Contributo alla flora vascolare urbana di Chieti (Abruzzo). Webbia 56: 343–378.

Martini F (2006) La flora vascolare spontanea della città di Trieste. Webbia 61: 57–94.

Mele C, Annese B, Albano A, Marchiori S (2002) Contributo alla flora e vegetazione del centro storico di Lecce (Puglia-Italia). Informatore Botanico Italiano 34: 91–104.

Pirone G, Ferretti C (1999) Flora e vegetazione spontanee della città di Pescara (Abruzzo, Italia). Fitosociologia 36: 111–155.

Poli Marchese E, Grillo M, Maugeri G (1989) Investigation of spontaneous urban flora of the city of Catania (Sicily). Braun-Blanquetia 3: 137–142.

Ricotta C, La Sorte FA, Pyšek P, Rapson GL, Celesti-Grapow L, Thompson K (2009) Phyloecology of urban alien floras. J Ecol 97: 1243-1251.

Verona V, Condolini A, Cenci CA, Pagiotti R, Menghini L (2004) La flora spontanea della città di Udine. Informatore Botanico Italiano 36: 363–399.
